# Transient ischemic dilation ratio by [^13^N]NH_3_ PET/CT in patients with variable extent of myocardial ischemia: relevance of software package and quantification method

**DOI:** 10.1007/s10554-025-03468-w

**Published:** 2025-07-18

**Authors:** Tonantzin Samara Martínez-Lucio, Sergiy V. Lazarenko, Oscar Isaac Mendoza-Ibañez, Remco J. J. Knol, Andrea G. Monroy-Gonzalez, Friso van der Zant, Charalampos Tsoumpas, Riemer H. J. A. Slart

**Affiliations:** 1 Department of Nuclear Medicine and Molecular Imaging, University of Groningen and University Medical Center Groningen, Groningen, The Netherlands; 2 Department of Nuclear Medicine, Northwest Clinics Alkmaar, Alkmaar, The Netherlands; 3 Faculty of Science and Technology, Biomedical Photonic Imaging group, University of Twente, Enschede, The Netherlands

**Keywords:** Positron emission tomography, Transient ischemic dilation, Coronary artery disease, Left ventricular ejection fraction reserve, Myocardial flow reserve

## Abstract

Transient ischemic dilation has been conventionally calculated from the static images, and has been scarcely investigated by PET/CT. Therefore, we aim to evaluate the impact of the quantification method and software package on TID ratio by [^13^N]NH_3_ PET/CT; moreover, perform a comprehensive evaluation of TID in normal-perfusion and myocardial ischemic patients. Retrospective analysis of 125 [^13^N]NH_3_ PET/CT scans with Corridor 4DM and QPS/QGS software packages. TID ratio was calculated from static and gated left ventricular volumes. Comparisons were performed according to ischemic extent and software package. Correlations with LV function and perfusion variables were assessed. TID ratios were significantly higher in ischemic patients (*p* < 0.05), except for TID gated EDV from QPS/QGS. TID static and TID gated ESV from QPS/QGS were different between the very severe ischemic subgroup and the group without ischemia (*p* = 0.0003). TID gated ESV from Corridor 4DM was higher in moderate, severe, and very severe ischemic subgroups than the normal-perfusion group (*p* = 0.02). TID gated ESV presented strong inverse correlations with myocardial flow reserve (MFR) in the very severe ischemic subgroup with Corridor 4DM, and with ejection fraction (LVEF) reserve, independent of the software and percentage of ischemic extent. Upper limits for TID static, ESV, and EDV with QPS/QGS were 1.17, 1.57, and 1.3, respectively. TID static, ESV, and EDV upper limits with Corridor4DM were 1.25, 1.29, and 1.21, respectively. TID ratios from [^13^N]NH_3_ PET/CT are higher in ischemic than in normal-perfusion patients and related to ischemic extent. TID ratios correlated strongly with LVEF reserve and MFR. The processing software and metric quantification method may influence the TID ratio.

**Supplementary Information:**

The online version contains supplementary material available at 10.1007/s10554-025-03468-w.

## Introduction

Myocardial perfusion imaging (MPI) by positron emission tomography (PET) has become an important alternative to single photon emission computed tomography (SPECT) in the evaluation of coronary artery disease (CAD), due to its superior diagnostic accuracy, lower radiation dose, shorter acquisition time, and ability to quantify absolute myocardial blood flow (MBF) [1–4]. Despite the increased availability and clinical use of PET/CT, some validated and generally accepted parameters on MPI SPECT have only been scarcely investigated in PET. This has been the case for some parameters that provide information about left ventricular (LV) geometry [5], as well as other LV function parameters, such as the transient ischemic dilation (TID) ratio. TID ratio reflects a significant enlargement of the LV cavity on stress images in comparison with rest images, calculated by the ratio between stress and rest endocardial volumes conventionally from static non-gated images; however, it can also be derived from the volumes acquired from the gated series [6, 7].

Two mechanisms have been proposed as the cause of TID: the first is a true LV enlargement due to myocardial stunning or transient post-ischemic myocardial dysfunction. The second is an apparent dilation caused by extensive subendocardial ischemia. *Hung et al.* described in 2005 that, according to the theory of subendocardial ischemia, the degree of enlargement for EDV and ESV should be the same in patients with TID. Contrary, a true dilation caused by ischemic stunning causes a reduction of LV function, enlargement of the cavity, especially on the ESV [6].

TID ratio on SPECT is positioned as an established marker of severe and extensive ischemia, and a predictor of cardiac events. The reference limit of the TID ratio is wide-ranging. Studies evaluating its diagnostic performance state a TID range from 1.012 to 1.36, while prognostic studies report a range from 1.005 to 1.22 in SPECT [8]. This variation is attributable to the imaging protocol, radiotracer, and type of stress used, among other factors [8]. TID has scarcely been investigated with PET/CT. In studies performed with ^82^Rb, a TID threshold of 1.13–1.15 has been proposed by evaluating its accuracy in the diagnosis of CAD, and an association has been reported between abnormal TID and extensive left ventricular dysfunction, ischemic compromise, and reduced global flow reserve [9, 10]. In addition, a recent association of cardiac death risk was found in patients with a TID ratio larger than 1.07 [11]. Two research groups have evaluated TID on [^13^N]NH_3_ PET/CT MPI. Harland et al. assessed the feasibility of an exercise treadmill in thirty-three patients with suspected CAD and reported a mean summed stress score (SSS) of 4.5 ± 5.7 and a TID ratio of 1.01 ± 0.1, however, TID was not the main objective of the research [12]. More recently, *Jia et al.* suggested that a TID ratio ≥ 1.03 may identify those patients with non-obstructive CAD who have a high risk of major adverse cardiac events.

Given the relevance of TID, the absence of a definite underlying mechanism, the reported variability of its threshold, and the scarce information in PET/CT, the objective of this study was to evaluate the impact of quantification method and software package on TID ratio by [^13^N]NH_3_ MPI PET/CT, moreover, perform a comprehensive evaluation of TID in normal-perfusion and myocardial ischemic patients.

## Methods

### Study population

In total, 125 patients with suspected CAD who underwent ECG-gated [^13^N]NH_3_ at Northwest Clinics Alkmaar in Alkmaar, The Netherlands, between March 2014 and May 2020 were retrospectively selected. In Online Resource Fig. 1 a flowchart describes in detail the inclusion of the patients.

**Fig. 1:**
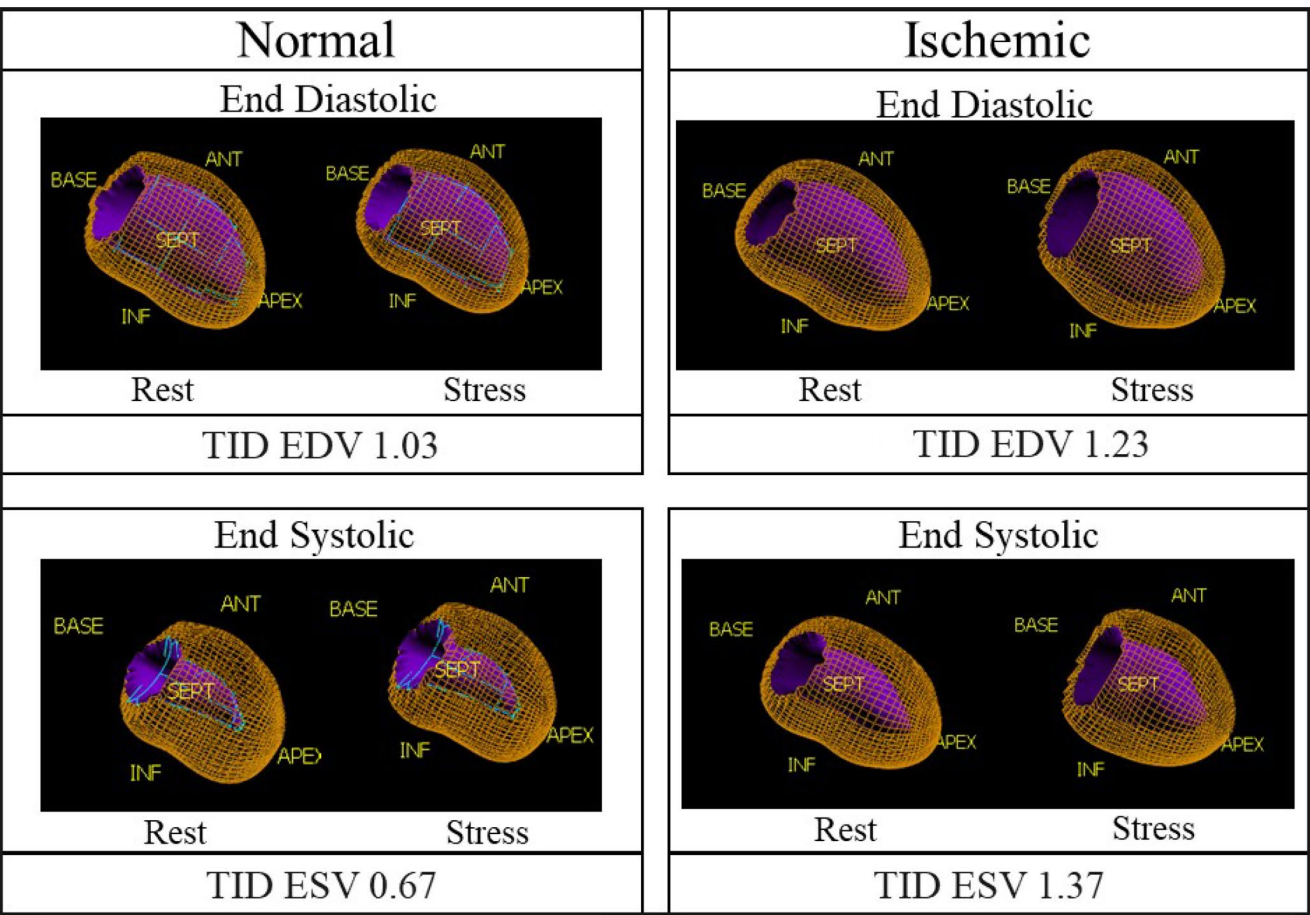
Representation of the LV surface from two patients. Wireframe surface corresponds to epicardium and shaded surface to endocardium. Note how TID ratio is higher in the ischemic patient, regardless of ES or ED phase. The conventional TID ratio from static images was 0.92 in the patient with normal perfusion and 1.16 in the patient with ischemia

The study population included two types of patients: normal-perfusion patients and patients with myocardial ischemia. The normal-perfusion group was included by medical consensus between nuclear physicians based on no prior history of CAD, and normal perfusion (semi-quantitative scores lower than 5, stress myocardial blood flow [MBF] higher than 1.85 ml/g/min) and/or coronary flow reserve [CFR] higher than 2.0) by PET/CT [13]. For the ischemic patients, the inclusion criteria consisted of a consensus diagnosis of myocardial ischemia by nuclear physicians based on the clinical history and PET/CT results compatible with ischemia in the LV-wall by visual defects, ischemic burden calculated with summed difference score (SDS) higher than 5%, low-stress MBF (< 1.85 ml/g/min) and/or low CFR (< 2.0) [13]. Patients with signs of myocardial infarction in the ECG-gated [^13^N]NH_3_ PET/CT (fixed visual defects combined with low rest MBF) were excluded.

Demographic information such as age, sex, body mass index, and cardiac risk factors, specifically hypertension, diabetes mellitus, hypercholesterolemia, smoking, and history of prior percutaneous coronary intervention and coronary artery bypass graft surgery, was retrieved from the clinical files and saved anonymously. Information about the presence of calcium in the coronaries from the low-dose CT and vasodilation used for the stress scan was retrieved.

The local scientific board approved this retrospective study, and the approval of the local ethical committee was waived since this study does not fall within the scope of the Dutch Medical Research Involving Human Subjects Act (Sect. 1.b WMO, 26th February, 1998). All patients gave written informed consent to use their anonymized data.

### PET/CT

For the acquisition of ECG-gated [^13^N]NH_3_ myocardial PET/CT, patients were instructed to refrain from caffeine and xanthine consumption 24 h before the scan procedure. All images were acquired in listmode using a Biograph 16 TruePoint PET/CT system (Siemens Healthineers, Erlangen, Germany), which consists of a 16-slice CT component and a 21.6 cm axial field of view PET scanner.

The imaging process started with a topogram acquisition (110 kVp, 25 mAs) used for patient positioning. Afterward, a low-dose CT scan was acquired (130 kVp, 25 mAs) for attenuation correction. For the PET acquisition, patients were injected with 300 MBq of [^13^N]NH_3_ (3 ml [^13^N]NH_3,_ at a rate of 0.4 ml/s followed by 17 ml NaCl and flushed with 20 ml NaCl at a rate of 20 ml/s) and a 12-minute rest imaging started simultaneously with the infusion. Stress was induced by intravenous administration of either adenosine (0.14 mg/kg/min for 6 min) or 400 µg regadenoson (single bolus), through a second line, immediately after the acquisition of rest images [14]. A 12-minute stress imaging started one minute after the end of the rest acquisition. Three minutes after the administration of the pharmacologic stressor, a second dose of 400 MBq of [^13^N]NH_3_ (3 ml [^13^N]NH_3,_ at a rate of 0.4 ml/s) was infused.

Static, 16-bin ECG-gated, and dynamic images were generated from listmode data. three-dimensions (3D) attenuation-weighted ordered subsets expectation maximization (OSEM3D) reconstruction with a 168 × 168 matrix, zoom of two, pixel spacing 2.04 mm × 2.04 mm, slice thickness of three mm, Gaussian filter of five mm in full width at half maximum, two iterations, 21 subsets for gated and dynamic images. TrueX OSEM3D reconstruction algorithm with point-spread function correction, zoom of two, matrix of 256 × 256, pixel spacing 1.34 mm x 1.34 mm, slice thickness of three mm, Gaussian filter of four mm, four iterations, and eight subsets was performed for the generation of the static images.

Residual activity correction was applied for dynamic and static images by excluding the interference of residual [^13^N]NH_3_ activity from the rest acquisition to the stress acquisition. This correction was performed with a method integrated into the Syngo MBF software package (Siemens Healthineers) [14].

The correct alignment between PET and CT was evaluated by experienced technologists and in case of misalignment, a correction was performed manually before starting the final reconstruction process.

Dynamic rest images were reconstructed from the first 10 min of the rest acquisition data, using 25 frames (1 × 10 s, 12 × 5 s, 2 × 10 s, 7 × 30 s, 2 × 60 s, 1 × 180 s) while dynamic stress images were reconstructed using the 10.5 min of data from the stress acquisition after a delay of 90 s, using 26 frames (1 × 30 s, 1 × 10 s, 12 × 5 s, 2 × 10 s, 7 × 30 s, 2 × 60, 1 × 180 s). To allow blood pool clearance of [^13^N]NH_3_, static and gated rest images were reconstructed from 2.5 min to 12 min, and stress images were reconstructed from 4.5 min to 12 min [15].

Static, dynamic, and 16-bin ECG-gated images from the selected patients were retrieved and analyzed with previously validated software packages, QGS/QPS 2017 (Cedars-Sinai Medical Center, Los Angeles, CA) and Corridor4DM (INVIA Medical Imaging Solutions, Ann Arbor, MI).

### Semiquantitative scores

Static images were processed with both software packages, however, only QPS/QGS parameters were used for classifying the patients. Visual semi-quantitative analysis was performed with the 17-segment model (AHA) and scored with a scale of five points (0–4) in summed rest score (SRS), summed stress score (SSS), and SDS. The percentage of ischemic extent was calculated using the SDS from QPS/QGS software, dividing SDS by the maximum possible score (4 points × 17 segments = 68) and multiplying by 100. Ischemic patients were classified into four subgroups according to the percentage of ischemic extent to maintain a similar number of patients per group, as follows: mild: 5-9.9%, moderate: 10-14.9%, severe: 15-24.9%, and very severe: ≥25%.

### Quantitative analysis of myocardial blood flow

In addition to the semi-quantitative assessment, MBF in rest, stress, and CFR were automatically acquired from both software packages. These quantitative results were evaluated to integrate the diagnosis. As described by EANM guidelines, the cut-off values of 1.85 mL/min/g for MBF stress and 2.0 for CFR were used as a reference [13]. Furthermore, the association of MBF and CFR with TID ratios was evaluated, as the TID ratio has been reported to be higher in patients with severe ischemia.

### ECG-gated PET/CT

ECG-gated rest and stress images were processed with both software packages. The algorithm of QPS/QGS is based on a volume-based method, where the LV endocardium is estimated in 3D, and the LV volume is measured as the territory delimited by the valve plane and endocardium for every interval in the cardiac cycle [6, 16, 17]. Automatically generating the end-diastolic volume (EDV), end-systolic volume (ESV), stroke volume (SV), and ejection fraction (EF). In the case of Corridor4DM, a LV endocardial surface algorithm is used to calculate a LV volume curve, from which volumes and EF are computed. The Corridor4DM surface algorithm allows the mitral valve to move towards the apex as much as 20 mm during systole. Therefore, LVEF values tend to be slightly higher [18].

### Left ventricular volumes and ejection fraction

ESV corresponds to the blood volume remaining in the LV cavity at the end of the contraction. On the other hand, EDV refers to the maximum volume of blood during the relaxation phase. EF is calculated automatically by the software packages from the values of the volumes: LVEF(%) = (EDV– ESV)/EDV × 100. These parameters were used to integrate the diagnosis. Moreover, as the presence of ischemia has been associated with impaired LV function, especially with a high degree of ischemia, a correlation analysis was performed in the ischemic group and per subgroup, to explore the association between TID ratios, and LV volumes, the change in LV volumes (stress– rest), LVEF, LVEF reserve (stress– rest).

### Transient ischemic dilation

QPS/QGS and Corridor4DM use non-gated 3D volumes to automatically calculate TID static, which is defined as the ratio of the LV volumes at stress and rest.

Additionally, TID ESV and TID EDV were calculated manually from the values of ESV and EDV, respectively, by the ratio of these volumes at stress and rest. Figure 1 illustrates the parametric representations of the LV with QPS/QGS from two patients included in our cohort.

Previous studies have reported some variability in TID values due to diverse factors. Therefore, we evaluated the differences in TID ratios between software packages. A stepwise approach was selected to assess the differences in TID ratio across populations as it is a proven marker of ischemia. First, mean TID values were compared between normal and ischemic patients to evaluate the capacity of TID to distinguish between these two populations. Second, mean TID values were compared between the normal-perfusion group and four ischemic subgroups. This second step was chosen to evaluate differences in the TID ratio according to the ischemic extent. Finally, sixty-three normal-perfusion patients were used to determine TID normal limits according to different volumes and software packages.

**Fig. 2:**
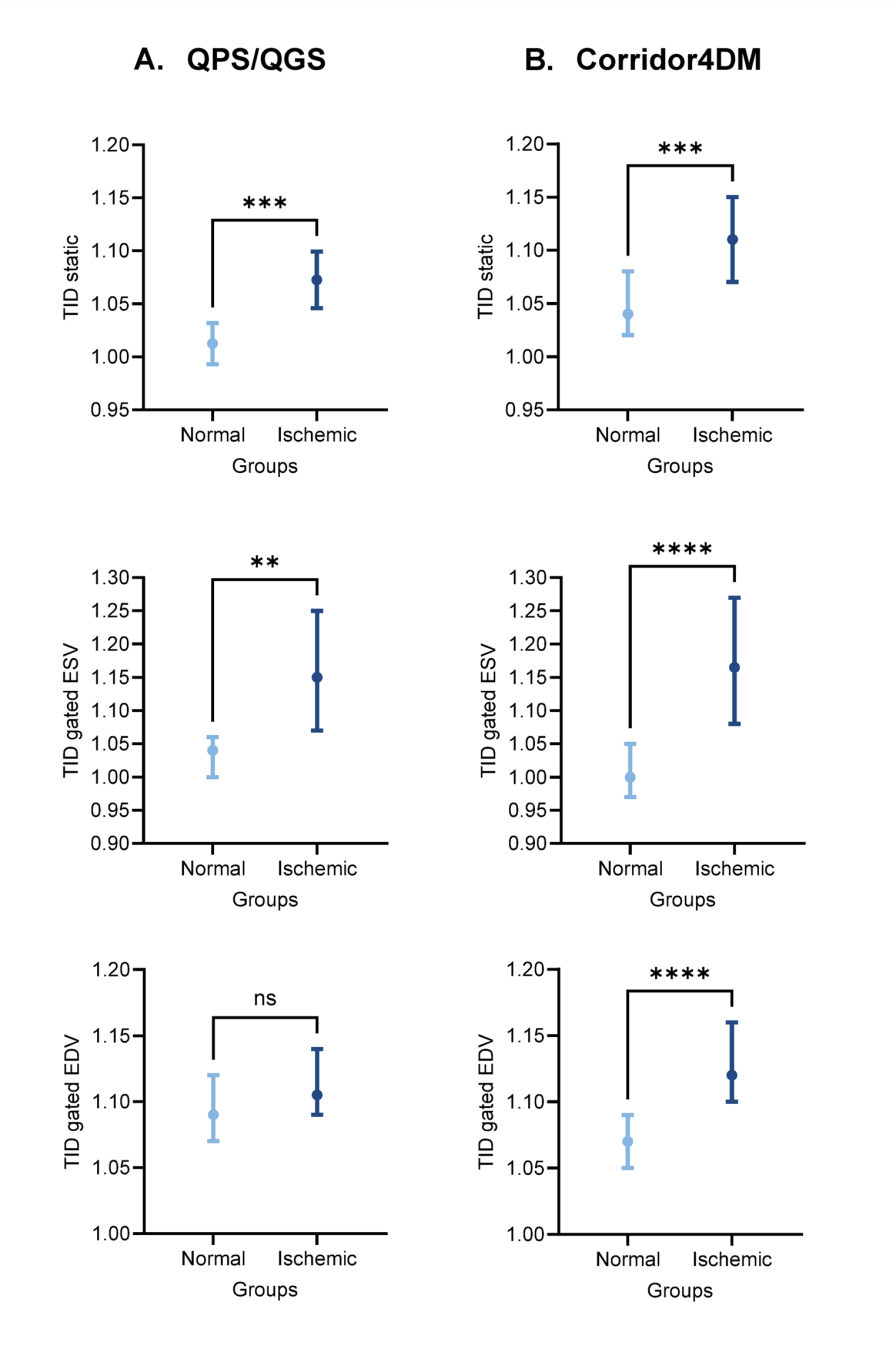
Comparison of TID ratios between normal-perfusion and ischemic groups with two software packages

### Statistical analysis

Normality tests were conducted using the Shapiro-Wilk test and Q-Q plot. Normally distributed categorical values were expressed using frequencies and percentages, whereas continuous values were presented as means ± standard deviation (SD). Baseline characteristics, risk factors, and cardiovascular history variables were compared as proportions with *χ*^2^. Comparisons between groups were performed using a paired or independent T-test.

Multiple comparisons between groups were evaluated using one-way ANOVA. To establish a control group, in this case, the normal-perfusion group, Dunnett’s test was selected as post hoc after finding a statistically significant one-way ANOVA. Bivariate Pearson’s correlation was used to investigate the strength and direction of association between continuous variables. Normal limits were calculated as mean + 2 SD as proposed by Shi et al. [9].

All statistical analyses were executed using IBM SPSS Statistics version 28.0 (Armonk, NY: IBM Corp., USA), where two-tailed *p*-values were considered statistically significant. Graphs were performed with GraphPad Prism 9.1.

## Results

### Patient characteristics

Our study consisted of 59% women, with a mean age of 67 ± 10 years. Women comprise 79.4% of the group of patients with normal perfusion, while they represent only 37.1% of the group of patients with myocardial ischaemia. Given the difference in the frequency of gender, although expected, a comparison of the variables of interest between men and women was conducted. Showing significant differences in SSS, SDS, MBF rest and stress, LV volumes, and LVEF, but not in SRS, CFR, and TID ratios. The complete results can be found in Online Resource Table 1. There was a high prevalence of hypertension (57.6%) and hypercholesterolemia (40%). The patients in the ischemic group were significantly older and predominantly men (56.5%). The prevalence of hypertension and hypercholesterolemia was considerably higher in the ischemic group in comparison with the normal-perfusion group. Regarding cardiovascular history, significant differences were found in all the characteristics as normal-perfusion patients did not present previous myocardial infarction, percutaneous coronary intervention, or coronary artery bypass graft surgery. Moreover, none of the patients in the normal-perfusion group presented calcium in the coronaries by attenuation correction CT done in the PET/CT. Adenosine was the predominantly used vasodilator in both groups, only four ischemic patients were stressed with regadenoson. The detailed clinical characteristics from all the patients and divided by normal-perfusion and ischemic groups are presented in Table 1. Additionally, these characteristics by ischemic subgroups are exposed in Online Resource Table 2.

**Table 1:** Baseline population characteristics

Variable		All *N* = 125	Normal-perfusion group *n* = 63	Ischemic group *n* = 62	*p* value
Age - mean years (SD)		67 (10)	63 (9)	71.08 (10)	< 0.001
Women - n (%)	74 (59.2)		50 (79.4)	23 (37.1)	< 0.001
Risk factors - n (%)					
Weight - mean kg (SD)	80.9 (15.4)		80 (16)	82.1 (15.2)	ns
Height - mean cm (SD)	170.2 (11.4)		168.9 (11)	171.4 (12.1)	ns
BMI - mean (SD)	27.9 (4.5)		27.9 (4.9)	27.9 (4.1)	ns
Smoking - n (%)	8 (6.4)		6 (9.5)	2 (3.2)	ns
Hypertension - n (%)	72 (57.6)		29 (46)	43 (69.4)	0.011
Diabetes mellitus - n (%)	18 (14.4)		6 (9.5)	9 (14.5)	ns
Hypercholesterolemia - n (%)	50 (40)		21 (33.3)	29 (46.8)	ns
Cardiovascular history - n (%)					
Prior myocardial infarction- n (%)		8 (6.4)	0 (0)	8 (12.9)	0.001
Prior PCI - n (%)	23 (18.4)		1 (1.6)	22 (35.5)	< 0.001
Prior CABG - n (%)		6 (4.8)	0 (0)	6 (9.7)	0.013
Calcium in coronaries - n (%)		58 (46.4)	3 (4.8)	55 (88.7)	< 0.001

**Table 2:** TID ratio calculated with different LV volumes and paired comparisons between software packages

Variable Mean (SD)	Normal-perfusion group *n* = 63			Ischemic group *n* = 62		
	QPS/QGS	Corridor4DM	*p* value	QPS/QGS	Corridor4DM	*p* value
Transient ischemic dilation static	1.01 (0.08)	1.05 (0.1)	< 0.001	1.07 (0.1)	1.1 (0.13)	< 0.001
Transient ischemic dilation gated end-systolic volume	1.09 (0.24)	1.03 (0.13)	0.005	1.21 (0.25)	1.24 (0.28)	ns
Transient ischemic dilation gated end-diastolic volume	1.1 (0.1)	1.07 (0.07)	< 0.001	1.11 (0.08)	1.13 (0.09)	ns

### Influence of calculation method and software on TID

Mean TID ratios and SD of normal-perfusion and ischemic groups are shown in Table 2. TID means were variable depending on the volume used for their calculation. The lowest TID mean (1.01 ± 0.08) corresponded to TID static in the normal-perfusion group acquired with QPS/QGS. The highest TID mean (1.24 ± 0.28) was TID ESV in the ischemic group acquired with Corridor4DM. On the paired comparison of TID ratios by software, all TID ratios were significantly different between QPS/QGS and Corridor4DM, except for TID gated ESV and TID gated EDV in the ischemic group.

### TID as a marker of severe ischemia

TID static, gated ESV, and gated EDV were compared between normal-perfusion and ischemic groups with independent T-tests. TID ratios from static and gated ESV acquired with QPS/QGS software from ischemic patients were significantly higher (*p* < 0.001 and *p* = 0.008). When TID ratios acquired with Corridor4DM software were compared between populations, the ischemic group presented ratios significantly higher than the normal-perfusion group regardless of the TID method. The TID ratio with the greatest difference between populations corresponded to TID gated ESV with a mean difference of 0.2, whereas TID static presented a mean difference of 0.07 and TID gated EDV of 0.06. The previous information is summarized and illustrated in Fig. 2.

**Fig. 3:**
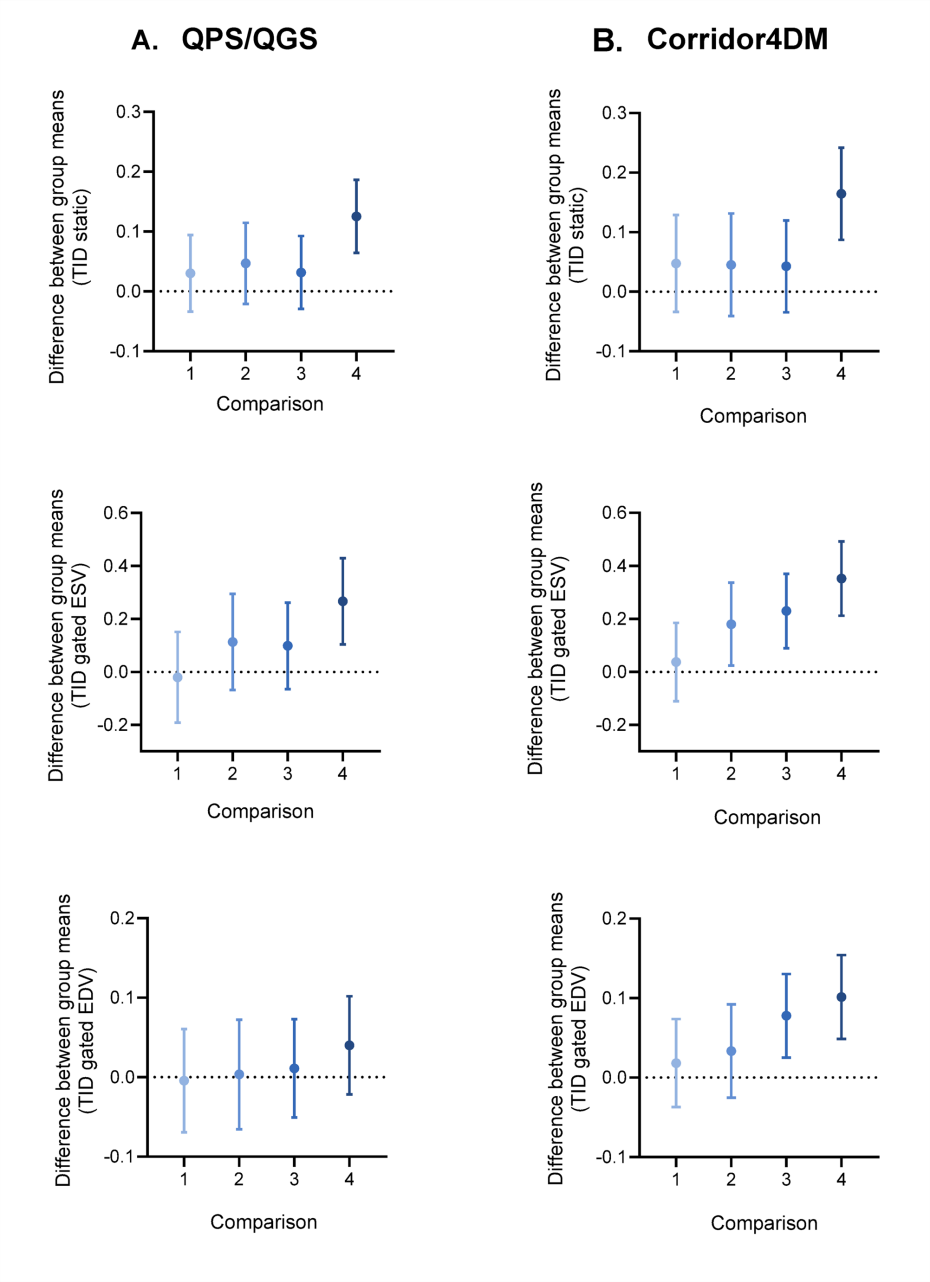
Multiple comparisons of TID ratios between ischemic subgroups and the normal-perfusion group (control). 1: mild, 2: moderate, 3: severe, 4: very severe ischemia

All the comparisons from TID ratios evaluated with one-way ANOVA were statistically significant except for TID gated EDV with QGS/QPS software. Subsequently, Dunnett’s Test was performed selecting the normal-perfusion group as the control. Figure 3 displays the mean TID ratios in patients categorized by varying extents of ischemia, as assessed using two different software packages. Regardless of the software used, TID increased with the extent of ischemia. More specifically, in QPS/QGS software analysis the mean TID static ratio was significantly different (*p* < 0.001) only between the normal-perfusion group (1.01 ± 0.08) and the very severe ischemic subgroup (1.14 ± 0.13). The same behavior was observed with TID gated ESV ratio, where the very severe ischemic subgroup (1.36 ± 0.33) was significantly different (*p* < 0.001) from the normal perfusion group (1.09 ± 0.24). Finally, TID gated EDV ratio did not present significant differences among the ischemic extent subgroups. In the analysis performed with Corridor4DM, the mean TID static ratio from the very severe ischemic subgroup (1.22 ± 0.17) was significantly different (*p* < 0.001) from the normal-perfusion group (1.05 ± 0.10). TID gated ESV means from the very severe (1.38 ± 0.33), severe (1.26 ± 0.27), and moderate subgroups (1.21 ± 0.24) were significantly higher (*p* < 0.05) in comparison with the normal-perfusion subgroup (1.03 ± 0.13). Finally, TID gated EDV ratio was significantly smaller (*p* = 0.001) in the normal-perfusion group (1.07 ± 0.07) when compared to severe (1.15 ± 0.10) and very severe ischemic subgroups (1.17 ± 0.09). Specific values of mean and SD of TID ratios per ischemic subgroup for both software packages are presented in Online Resource Tables 3 and 4.

**Table 3:** Functional variables of patients with and without myocardial ischemia

Variable Mean (SD)	QGS/QPS			Corridor4DM		
	Normal-perfusion group *n* = 63	Ischemic group *n* = 62	*p* value	Normal-perfusion group *n* = 63	Ischemic group *n* = 62	*p* value
End-systolic volume rest	30 (15)	32 (16)	0.4	42 (20)	45 (20)	ns
End-systolic volume stress	32 (16)	38 (20)	0.05	42 (19)	54 (25)	0.003
End-diastolic volume rest	99 (31)	102 (27)	0.6	112 (34)	116 (31)	ns
End-diastolic volume stress	108 (32)	113 (31)	0.4	119 (34)	131 (37)	0.05
Left ventricular ejection Fraction rest	71 (7.6)	70 (8)	0.3	64 (8)	63 (8)	ns
Left ventricular ejection Fraction stress	72 (8)	68 (8)	0.003	66 (7)	60 (8)	< 0.001

**Table 4:** Myocardial perfusion variables of patients with and without myocardial ischemia

Variable Mean (SD)	QGS/QPS			Corridor4DM		
	Normal-perfusion group *n* = 63	Ischemic group *n* = 62	*p* value	Normal-perfusion group *n* = 63	Ischemic group *n* = 62	*p* value
Summed rest score	0.54 (0.76)	1.16 (1.06)	< 0.001	2.62 (2.8)	2.69 (2.71)	ns
Summed stress score	1.4 (1.31)	13.74 (6.63)	< 0.001	3.03 (3.13)	13.9 (6.83)	< 0.001
Summed difference score	0.86 (1.05)	12.58 (6.61)	< 0.001	1.27 (1.72)	11.23 (6.88)	< 0.001
Global myocardial blood flow rest (ml/g/min)	0.90 (0.25)	0.87 (0.25)	0.6	0.89 (0.19)	0.84 (0.21)	ns
Global myocardial blood flow stress (ml/g/min)	2.38 (0.76)	1.70 (0.49)	< 0.001	2.76 (0.31)	2.01 (0.45)	< 0.001
Global coronary flow reserve	2.75 (0.87)	2.05 (0.74)	< 0.001	3.2 (0.71)	2.45 (0.62)	< 0.001

### Relationship between TID ratios and LV parameters

**Fig. 4:**
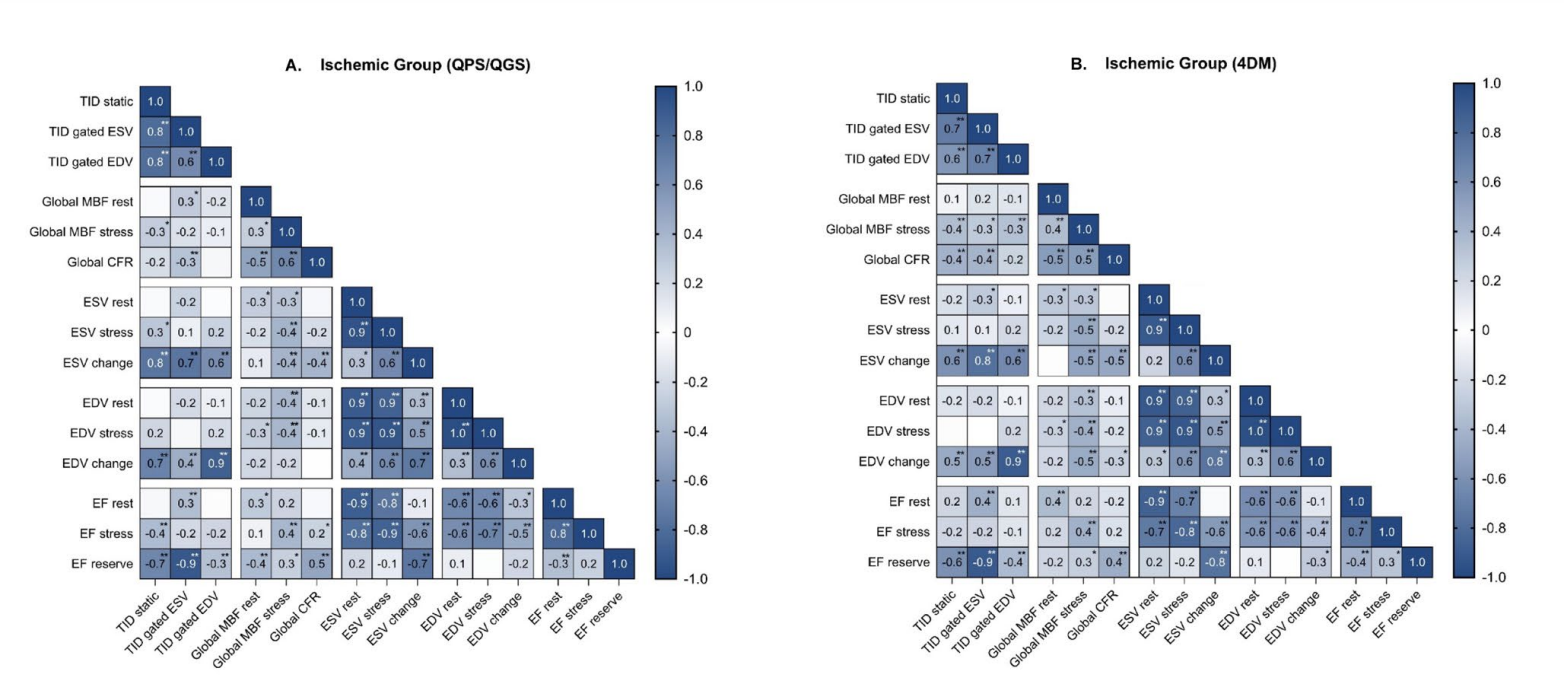
Correlation matrix depicting the relationships between TID ratios and parameters of LV function and perfusion from the ischemic group. (**A**) Analysis of values acquired with QPS/QGS. (**B**) Analysis of values acquired with Corridor4DM. MBF, myocardial blood flow; CFR, coronary flow reserve; ESV, end-systolic volume; EDV, end-diastolic volume; EF, ejection fraction. *p < 0.5, **p < 0.01

The results of the correlations in the ischemic group between TID ratios, and with the parameters of LV function are presented in Fig. 4. The values of volumes and EF for normal-perfusion and ischemic groups are presented in Table 3. LV volumes were higher in ischemic than in normal-perfusion patients. Consequently, EF was lower in the ischemic group. However, these differences were only significant for ESV stress and EF stress. Regarding the correlation analysis between TID ratios, TID static presented strong to very strong correlations with TID ESV and TID EDV with both software. Likewise, TID ESV and EDV presented strong correlations between them.

Only TID static was weakly but significantly correlated with ESV stress with QPS/QGS. Similarly, TID ESV presented a weak correlation with ESV rest. All TID ratios presented strong or very strong correlations with the change of ESV [∆ESV (stress-rest)], and moderate to very strong correlations with the change of EDV [∆EDV (stress-rest)], indistinct of the software. The analysis between TID ratios and EF revealed a weak to moderate association at rest with TID gated ESV in both software and an inverse moderate correlation between EF stress and TID static with QPS/QGS. EF reserve [∆EF (stress-rest)] presented inverse and very strong correlations with TID gated ESV. Moreover, the EF reserve was inverse and strongly correlated with TID static. Lastly, the EF reserve presented inverse weak to moderate correlations with TID gated EDV. The associations with EF reserve were indistinct of the software used. A detailed analysis was performed per ischemic subgroup and is presented as correlation matrices in Online Resource Fig. 2–5.

### Relationship between TID ratios and myocardial perfusion parameters

Correlation matrices in Figure 4 illustrate the relationship of TID ratios with myocardial perfusion parameters. Additionally, Table 4 outlines the results of SRS, SSS, SDS, MBF, and CFR by group and software. The ischemic group presented significantly higher SSS and SDS than the normal-perfusion group. In contrast, MBF stress and CFR were significantly lower in the ischemic group. The analysis with QPS/QGS global MBF at rest presented a weak correlation with TID gated ESV. Global MBF stress acquired with the same software correlated inversely with TID static, global CFR presented an inverse weak correlation with TID gated ESV. On the other hand, in the analysis with Corridor4DM global MBF rest did not correlate significantly with any TID ratio, whereas global MBF stress demonstrated inverse weak-moderate correlations with all TID ratios. Although there was no correlation between global CFR and TID EDV, the inverse moderate associations with TID static and TID ESV were significant. A detailed analysis was performed per ischemic subgroup and is presented as correlation matrices in Online Resource Fig. 2–5.

### Normal limits of TID in [^13^N]NH_3_

Six TID ratio thresholds were calculated by mean + 2 SD from the 63 normal-perfusion patients. Table 5 displays the TID thresholds from the two software packages. The lowest limit is TID static of 1.17, and the highest is TID ESV of 1.57, both calculated with QPS/QGS.

**Table 5:** TID thresholds with two software packages

		QPS/QGS	Corridor4DM
Transient ischemic dilation static		1.17	1.25
Transient ischemic dilation gated end-systolic volume	1.57		1.29
Transient ischemic dilation gated end-diastolic volume		1.3	1.21

## Discussion

The present study was designed to determine the impact of software package and LV volumes on the value of TID in [^13^N]NH_3_ MPI PET/CT.

Our findings demonstrate that the TID ratio is variable as it is calculated with LV volumes from different images (static non-gated, and gated end-systolic, and end-diastolic), which is supported by the following results. In the head-to-head comparison between software packages, all TID means were significantly different between QPS/QGS and Corridor4DM, except for TID gated ESV and EDV in the group with ischemic patients. One explanation could be that for the static non-gated reconstruction, the images contain counts from all cardiac cycles with a visual appearance similar to end-diastolic images. Resulting in a different value of the LV cavity volume compared to the final volume at end diastole and end systole [6, 17, 19]. Another finding that highlights the importance of the software selected for quantification was that TID gated EDV was significantly different between ischemic patients and normal-perfusion patients with Corridor4DM but not with QPS/QGS. Hu et al. have already highlighted the importance of software in the computation of TID [20]. Software packages determine LV volumes using different methods or calculate the TID ratio with specific algorithms. In this regard, Corridor 4DM describes that its surface algorithm differs from other software packages in the handling of the basal plane motion, allowing a movement of the mitral valve as much as 20 mm towards the apex [18]. 

It has been previously shown in SPECT, that the TID ratio can vary depending on the type of stressor used for vasodilation, the acquisition protocol, and the radiotracer used [20, 21]. In a systematic review and meta-analysis from Alama et al.., the ratio above which TID was diagnosed ranged from 1.012 to 1.36, being twelve out of the thirteen included studies performed with SPECT. Thus, imaging modality, radiotracer, type of stress, and software should be considered when evaluating TID.

TID gated ESV presented higher mean differences between normal and abnormal patients (i.e., 0.12 in QPS/QGS, 0.21 in 4DM) than TID static (i.e., 0.06 in QPS/QGS, 0.08 in 4DM) and TID gated EDV (i.e., 0.02 in QPS/QGS, 0.06 in 4DM). Moreover, TID gated ESV and EDV increase steadily as the ischemic extent is higher, more noticeable with Corridor4DM. Despite the increasing pattern of TID with the ischemic extent, only significant differences with TID gated ESV were found between the normal-perfusion and very severe ischemic subgroup in QPS/QGS. Whereas with 4DM, TID static was significantly higher in the subgroup with very severe ischemia, TID gated EDV was significantly higher in the severe and very severe ischemic subgroup, and TID gated ESV was significantly higher from the moderate to very severe ischemic subgroups in comparison with the normal-perfusion group. A possible explanation for a higher difference in TID gated ESV than with TID static, between normal-perfusion and ischemic patients could be that gated images may be more accurate in the detection of LV contours than static images, as the image is taken only from one cardiac phase, end-systole.

The noticeable difference in TID means between normal-perfusion and very severe ischemic subgroup, may reflect post-stress stunning due to the stress-induced ischemia, which can be expected in patients with more severe disease. In this regard, in 2000 Peace et al.. reported TID obtained by ^99m^Tc-tetrofosmin SPECT as an indicator of severe CAD [22]. Later, in 2004, Abidov et al.. reported that an abnormal TID ratio correlated better with multivessel CAD than multiple perfusion abnormalities or summed perfusion scores [23].

Despite no correlations being found between TID ratios and ESV or EDV at rest and stress, ∆ESV and ∆EDV showed moderate to very strong correlations with TID ratios. These correlations were observed with both software tools.Almost no correlations were observed between TID ratios and EF. However, TID ratios predominantly TID gated ESV presented inverse and moderate to very strong correlations with ∆EF, with both software packages. Regarding the relationship with perfusion parameters, only Global CFR presented moderate inverse correlations with TID ESV in both software packages. All these results from correlation analysis are highly consistent with those of Rischpler et al.. who evaluated with ^82^Rb, the correlations of TID with parameters of function and perfusion in a high-risk group (CFR < 1.98). First, they reported no correlation between LV volumes and TID. However, both ∆ESV and ∆EDV were significantly correlated with TID (∆ESV: *R*^*2*^ = 0.23 and ∆EDV: *R*^*2*^ = 0.17; both *p* < 0.0001). Second, they describe no correlations with EF but report a negative correlation between TID and EF reserve (*R*^*2*^ = 0.06; *p* < 0.0005). Third, they describe a weak but significant correlation between CFR and TID (*R*^*2*^ = −0.04; *p* < 0.003) [10]. In our very severe ischemic subgroup, similar in characteristics to the high-risk group in Rischpler et al.., the correlations between TID ratios and ∆ESV, ∆EDV, ∆EF, and CFR were stronger in both software packages.

Recently, Jia et al.. published that a TID ratio ≥ 1.03 was associated with an increased risk of major adverse cardiac events (MACE) (log-rank *p* = 0.024) in 131 patients with non-obstructive CAD evaluated with [^13^N]NH_3_ PET/CT [24]. A cut-off value of 1.03 seems low for what has been previously described both with SPECT and PET. Only De Winter et al.. have proposed a low threshold of 1.005, although in ^99m^Tc SPECT [25]. Despite the differences in study populations and thresholds, our results are consistent with those from Jia et al., as the TID-abnormal group showed a significantly lower ∆EF reserve, while ∆ESV and ∆EDV were significantly higher compared to the TID-normal group.

In the current study, we calculated upper thresholds according to the volumes and software used for calculating TID ratios, ranging from 1.17 (TID static, QPS/QGS) to 1.57 (TID gated ESV, QPS/QGS). These thresholds are higher than those previously proposed in literature, especially if we focus on the thresholds for PET/CT. Only TID static acquired with QPS/QGS is close to the limit of 1.15 for ^82^Rb. Interestingly, only the mean TID gated ESV (1.38) from the group with very severe ischemia was higher than the proposed threshold of TID gated ESV (1.29) with Corridor4DM. All previous publications have measured TID based on the endocardial volumes derived from static acquisitions (TID static) [6, 9, 10, 16, 21, 26], in consequence, the proposed thresholds had been developed only using this methodology. In the case of PET/CT, two studies with ^82^Rb have proposed different thresholds for diagnosing TID: 1.13 [10] and 1.15 [9]. In our study, a mean TID ratio above these proposed thresholds was observed only with TID gated ESV in the ischemic group, irrespective of the software. Contrary to expectations, the mean TID static ratio from ischemic patients acquired from both software packages was lower than the thresholds proposed with ^82^Rb. Nonetheless, TID ratios in the subgroup of patients with very severe ischemia were higher than 1.13.

Although the TID ratio is traditionally calculated from static non-gated images, previous literature highlights the relevance of ESV. Our findings suggest that TID gated ESV could perform better in differentiating ischemic patients and that additional research regarding TID must consider this fact and evaluate if it can lead to more homogenous thresholds or better diagnostic and prognostic capabilities. Since 1994, Van Tosh et al., in a study combining Tl-201 SPECT and echocardiography, reported that patients without TID show a decrease in end-systolic area after stress while patients with TID presented an increase in end-systolic area and no significant differences in end-diastolic area for both groups [27]. Bestetti et al.. investigated ischemic stunning by stress-rest gated ^99m^Tc SPECT and showed a significant relationship between post-stress LVEF impairment and post-stress end-systolic ventricular dilation, and no correlation with an increased EDV [28]. Finally, Hung et al., using TL-201 gated SPECT, reported that ischemic stunning causes a depression of LV function and enlargement of ESV, resulting in TID on ungated perfusion images [6]. The impact of the increase in ESV in the calculation of TID may be the reason for the remarkable behavior of TID gated ESV in our study. This raises the question of whether the averaged non-gated LV volumes should still be used to calculate TID. Perhaps, when TID is derived from gated volume measurements, it would be possible to identify the underlying etiology of TID and enable the appropriate treatment.

### Limitations

Our study has three main limitations: First, the retrospective design. Second the small sample size, which becomes smaller when classifying ischemic patients according to their percentage of ischemia. This limits the generalizability of the results. And third, the lack of angiography or computed tomography angiography, which means that there was no information about coronary obstruction considered for the inclusion, classification of patients, or as a gold standard for testing diagnostic accuracy of the proposed TID thresholds. However, the presence of calcium in the coronaries was detected from the attenuation correction CT of the myocardial perfusion PET/CT. An additional limitation was the lack of prognostic information, in consequence, it was not possible to evaluate the TID prediction value of MACE.

### Future perspectives

Given the variability in the proposed limits for TID, research with larger groups of patients is needed to obtain clearer limits. Prospective studies are important to investigate the pathophysiological cause(s), applying important techniques such as motion correction for the acquisition of accurate images.Recently, a study published by *Patel et al.* evaluated the prognostic value in 9,878 patients with LVEF > 40% with normal and abnormal perfusion and demonstrated that TID offers a predictive value independent of other perfusion and non-perfusion high-risk markers, as ∆LVEF and CFR. Further studies are required to elicit a threshold for TID with [^13^N]NH_3_ in the setting of the prognostic accuracy of TID in PET/CT in diverse populations such as microvascular disease and three-vessel disease. As known from SPECT MPI, the next step is to investigate the differences in the TID ratio between pharmacologic vasodilators, for instance between adenosine and regadenoson [29].

### New knowledge gained and clinical implications

TID ratios calculated by [^13^N]NH_3_ PET/CT are related to ischemic disease and, more specifically, to ischemic extent. The presence of significant differences in TID ratios between ischemic and normal-perfusion populations and even between ischemic extents depends on the gated volumes, which are used for the calculation of the TID ratio, with gated ESV as most optimal. Additionally, the behavior in each software is different and may imply the clinical significance of the TID ratio.

## Conclusion(s)

TID ratios measured with [^13^N]NH_3_ PET/CT are higher in ischemic than in normal-perfusion patients and are related to ischemic extent. Diverse thresholds were proposed according to the volume and software used for their calculation. However, only a TID gated ESV mean from the group with very severe ischemia was higher than the proposed threshold of TID gated ESV (1.29) with Corridor4DM. TID ratios are strongly correlated with some parameters of LV function and perfusion, such as LVEF reserve and CFR, which are also important markers of ischemic extent. The processing software and metric quantification method may influence TID ratio values.

## Electronic supplementary material

Below is the link to the electronic supplementary material.

Supplementary Material. Transient ischemic dilation ratio by [13N]NH3 PETCT

## Data Availability

No datasets were generated or analysed during the current study.

## Abbreviations

CAD: Coronary artery disease
CFR: Coronary flow reserve
EDV: End diastolic volume
ESV: End systolic volume
IHD: Ischemic heart diseases
LVEF: Left ventricular ejection fraction
MBF: Myocardial blood flow
MPI: Myocardial perfusion imaging
PET/CT: Positron emission tomography/computed tomography
SPECT: Single photon emission tomography
SV: Stroke volume
SDS: Summed difference score
SRS: Summed rest score
SSS: Summed stress score
TID: Transient ischemic dilation
