# Supplementary material for: Transient ischemic dilation ratio by [13N]NH3 PET/CT in patients with variable extent of myocardial ischemia: relevance of software package and quantification method
Source: Int J Cardiovasc Imaging. 2025 Jul 18;41(8):1605–17. doi: 10.1007/s10554-025-03468-w (PMC12396988; doi:10.1007/s10554-025-03468-w)
Supplement: Supplementary file 1 — Supplementary Material. Transient ischemic dilation ratio by [13N]NH3 PETCT [file 10554_2025_3468_MOESM1_ESM.docx]

**Transient ischemic dilation ratio by [^13^N]NH_3_ PET/CT in patients with variable extent of myocardial ischemia. Relevance of software package and quantification method.**

**The International Journal of Cardiovascular Imaging**

Tonantzin Samara Martínez-Lucio TS, MD^1^, Sergiy V. Lazarenko, PhD^2^, Oscar Isaac Mendoza-Ibañez, MD^1^, Remco J.J. Knol, MD, PhD^2^, Andrea G Monroy-Gonzalez, MD, PhD^1^, Friso van der Zant, MD, PhD^2^, Charalampos Tsoumpas, PhD^1^, Sergiy V. Lazarenko, PhD^2^, Riemer H.J.A. Slart, MD, PhD^1,3^

**Corresponding author:**

Tonantzin Samara Martínez-Lucio, MD

University of Groningen and University Medical Centre Groningen, Department of Nuclear Medicine and Molecular Imaging, Groningen, The Netherlands.

E-mail: [t.s.martinez.lucio@umcg.nl](mailto:t.s.martinez.lucio@umcg.nl)

**Table 1.** Myocardial perfusion and LV function variables compared by gender

| **Variable**  **Mean (SD)** | **QPS/QGS** | | |  | **Corridor4DM** | | |
| --- | --- | --- | --- | --- | --- | --- | --- |
|  | **Female** | **Male** | ***p* value** |  | **Female** | **Male** | ***p* value** |
| **Summed rest score** | 0.82 (0.92) | 0.88 (1.04) | ns |  | 2.59 (2.63) | 2.75 (2.9) | ns |
| **Summed stress score** | 5.47 (7.09) | 10.4 (7.91) | <0.001 |  | 6.55 (7.25) | 11.06 (7.33) | <0.001 |
| **Summed difference score** | 4.64 (6.76) | 9.52 (7.7) | <0.001 |  | 4.62 (6.59) | 8.44 (7.14) | 0.002 |
| **Global myocardial blood flow rest (ml/g/min)** | 0.96 (0.26) | 0.78 (0.18) | <0.001 |  | 0.92 (0.21) | 0.79 (0.15) | <0.001 |
| **Global myocardial blood flow stress (ml/g/min)** | 2.25 (0.73) | 1.74 (0.6) | <0.001 |  | 2.6 (0.45) | 2.09 (0.52) | <0.001 |
| **Global coronary flow reserve** | 2.47 (0.93) | 2.3 (0.8) | ns |  | 2.91 (0.77) | 2.7 (0.75) | ns |
| **End-systolic volume rest** | 24.52 (11.87) | 40.4 (15.65) | <0.001 |  | 34.58 (15.39) | 55.88 (18.96) | <0.001 |
| **End-systolic volume stress** | 27.38 (11.97) | 45.33 (19.64) | <0.001 |  | 37.84 (15.03) | 62.65 (24.59) | <0.001 |
| **End-diastolic volume rest** | 87.84 (21.39) | 118.35 (28.73) | <0.001 |  | 99.58 (24.01) | 134.37 (32.42) | <0.001 |
| **End-diastolic volume stress** | 97.16 (22.38) | 129.88 (32.66) | <0.001 |  | 108.32 (24.51) | 148.77 (36.15) | <0.001 |
| **Left ventricular ejection fraction rest** | 73.12 (7.37) | 66.61 (6.71) | <0.001 |  | 66.41 (7.45) | 59 (7.09) | <0.001 |
| **Left ventricular ejection fraction stress** | 72.55 (7.63) | 66.02 (6.7) | <0.001 |  | 65.89 (7.6) | 58.69 (7.13) | <0.001 |
| **Transient ischemic dilation static** | 1.04 (0.1) | 1.05 (0.09) | ns |  | 1.09 (0.13) | 1.09 (0.12) | ns |
| **Transient ischemic dilation gated end-systolic volume** | 1.16 (0.29) | 1.13 (0.18) | ns |  | 1.13 (0.26) | 1.14 (0.22) | ns |
| **Transient ischemic dilation gated end-diastolic volume** | 1.11 (0.1) | 1.10 (0.07) | ns |  | 1.09 (0.08) | 1.11 (0.09) | ns |

**Table 2.** Baseline population characteristics by subgroup

| **Variable** | **None n = 63** | **Mild n = 15** | **Moderate n = 13** | **Severe n = 17** | **Very Severe n = 17** | ***p* value** |
| --- | --- | --- | --- | --- | --- | --- |
| **Age - mean years (SD)** | 63 (9) | 72 (9) | 73 (8) | 72 (11) | 69 (10) | <0.001 |
| **Women - n (%)** | 50 (79.4) | 6 (40) | 4 (30.8) | 6 (35.3) | 7 (41.2) | <0.001 |
| **Risk factors - n (%)** |  |  |  |  |  |  |
| **Weight - mean kg (SD)** | 80 (16) | 79.2 (11.2) | 83.4 (17.4) | 77.2 (15.3) | 88.3 (15.2) | ns |
| **Height - mean cm (SD)** | 168.9 (11) | 172.2 (11) | 171.9 (12.7) | 168.6 (11.6) | 173.2 (13.7) | ns |
| **BMI - mean (SD)** | 27.9 (4.9) | 26.8 (4.2) | 28.1 (4.1) | 26.9 (3.2) | 29.5 (4.7) | ns |
| **Smoking - n (%)** | 6 (9.5) | 1 (6.7) | 0 (0) | 1 (5.9) | 0 (0) | ns |
| **Hypertension - n (%)** | 29 (46) | 8 (53.3) | 7 (53.8) | 12 (70.6) | 16 (94.1) | 0.007 |
| **Diabetes mellitus - n (%)** | 6 (9.5) | 2 (13.3) | 3 (23.1) | 1 (5.9) | 3 (17.6) | ns |
| **Hypercholesterolemia - n (%)** | 21 (33.3) | 8 (53.3) | 5 (38.5) | 6 (35.3) | 10 (58.8) | ns |
| **Cardiovascular history - n (%)** |  |  |  |  |  |  |
| **Prior myocardial infarction- n (%)** | 0 (0) | 4 (26.7) | 3 (23.1) | 1 (5.9) | 0 (0) | <0.001 |
| **Prior PCI - n (%)** | 1 (1.6) | 6 (40) | 7 (53.8) | 5 (29.4) | 4 (23.5) | <0.001 |
| **Prior CABG - n (%)** | 0 (0) | 2 (13.3) | 2 (15.4) | 2 (11.8) | 0 (0) | 0.02 |
| **Calcium in coronaries - n (%)** | 3 (4.8) | 11 (73.3) | 10 (76.9) | 17 (100) | 17 (100) | <0.001 |

**Table 3.** Multiple comparison analysis of TID ratios according to the percentage of myocardial ischemia with Dunnett’s Test (QPS/QGS software)

| **QPS/QGS** | | | | |
| --- | --- | --- | --- | --- |
| **Variable** | **Ischemic groups** | **Control group** | **Mean Difference** | ***p* value** |
|  | **Mean (SD)** | **Mean (SD)** |  |  |
| **Transient ischemic dilation static** | Mild: 1.04 (0.08) | None: 1.01 (0.08) | 0.03 | ns |
|  | Moderate: 1.06 (0.08) |  | 0.05 | ns |
|  | Severe: 1.04 (0.08) |  | 0.03 | ns |
|  | Very Severe: 1.14 (0.13) |  | 0.13* | <0.001 |
| **Transient ischemic dilation gated end-systolic volume** | Mild: 1.07 (0.13) | None: 1.09 (0.24) | -0.02 | ns |
|  | Moderate: 1.20 (0.25) |  | 0.11 | ns |
|  | Severe: 1.19 (0.17) |  | 0.1 | ns |
|  | Very Severe: 1.36 (0.33) |  | 0.27* | <0.001 |
| **Transient ischemic dilation gated end-diastolic volume** | Mild: 1.10 (0.08) | None: 1.10 (0.10) | 0 | ns |
|  | Moderate: 1.19 (0.17) |  | 0.01 | ns |
|  | Severe: 1.11 (0.06) |  | 0.01 | ns |
|  | Very Severe: 1.14 (0.10) |  | 0.04 | ns |

**Table 4.** Multiple comparison analysis of TID ratios according to the percentage of myocardial ischemia with Dunnett’s Test (Corridor4DM software)

| **Corridor4DM** | | | | |
| --- | --- | --- | --- | --- |
| **Variable** | **Ischemic groups** | **Control group** | **Mean Difference** | ***p* value** |
|  | **Mean (SD)** | **Mean (SD)** |  |  |
| **Transient ischemic dilation static** | Mild: 1.10 (0.08) | None: 1.05 (0.10) | 0.05 | ns |
|  | Moderate: 1.10 (0.08) |  | 0.05 | ns |
|  | Severe: 1.10 (0.14) |  | 0.04 | ns |
|  | Very Severe: 1.22 (0.17) |  | 0.16* | <0.001 |
| **Transient ischemic dilation gated end-systolic volume** | Mild: 1.07 (0.15) | None: 1.03 (0.13) | 0.04 | ns |
|  | Moderate: 1.21 (0.24) |  | 0.18* | 0.02 |
|  | Severe: 1.26 (0.27) |  | 0.23* | <0.001 |
|  | Very Severe: 1.38 (0.33) |  | 0.35* | <0.001 |
| **Transient ischemic dilation gated end-diastolic volume** | Mild: 1.09 (0.08) | None: 1.07 (0.07) | 0.02 | ns |
|  | Moderate: 1.10 (0.07) |  | 0.03 | ns |
|  | Severe: 1.15 (0.10) |  | 0.08* | 0.001 |
|  | Very Severe: 1.17 (0.09) |  | 0.1* | <0.001 |

**
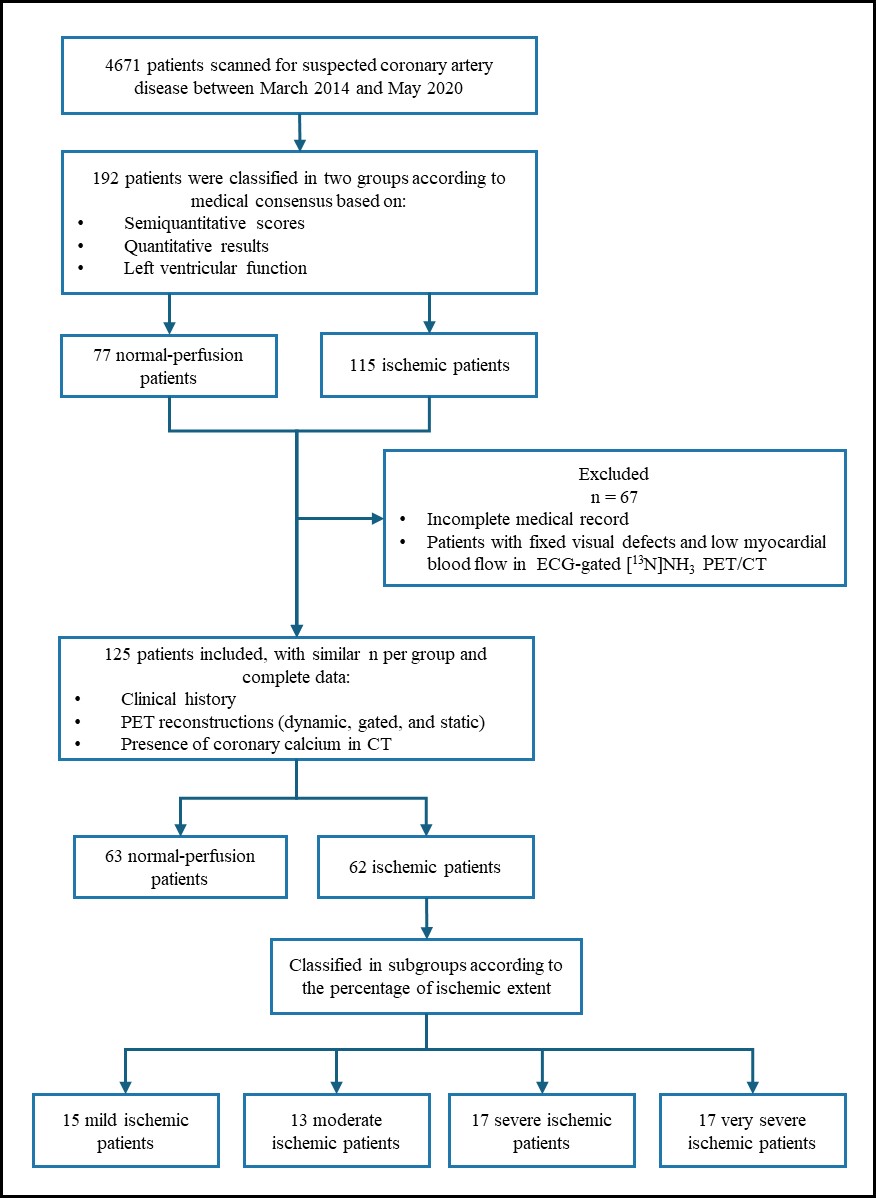
**

**Fig. 1** Flowchart showing the inclusion of patients


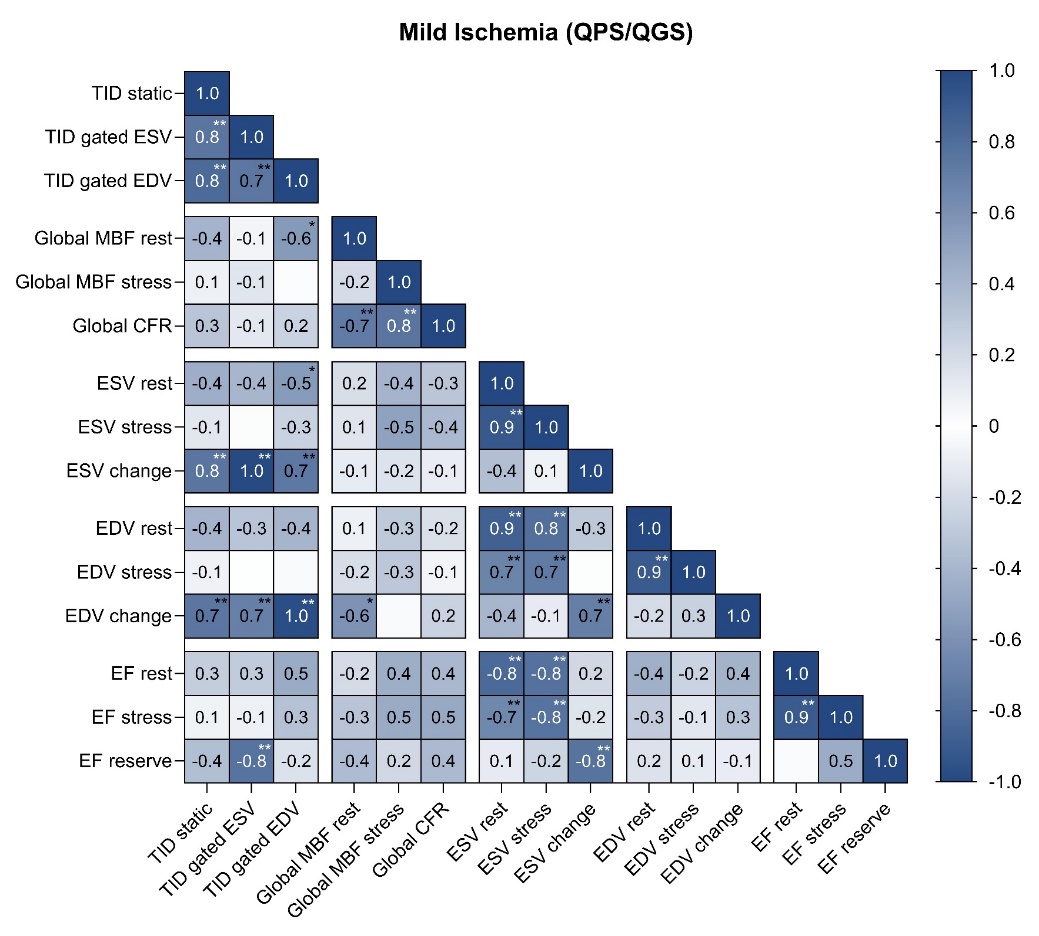


**a.**


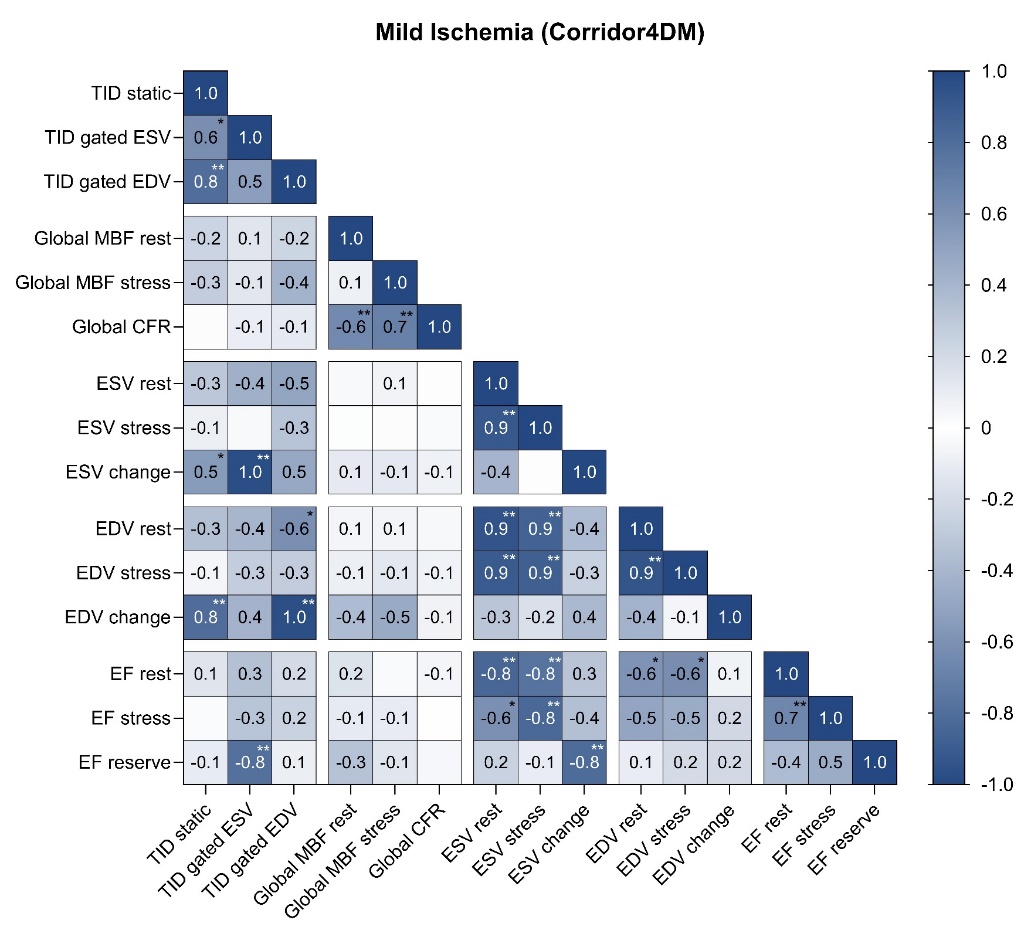


**b.**

**Fig. 2** Correlation matrix depicting the relationships between TID ratios and parameters of LV function and perfusion from the mild ischemic group. A. Analysis of values acquired with QPS/QGS. B. Analysis of values acquired with Corridor4DM. MBF, myocardial blood flow; CFR, coronary flow reserve; ESV, end-systolic volume; EDV, end-diastolic volume; EF, ejection fraction. **p*<0.5, ***p*<0.01.


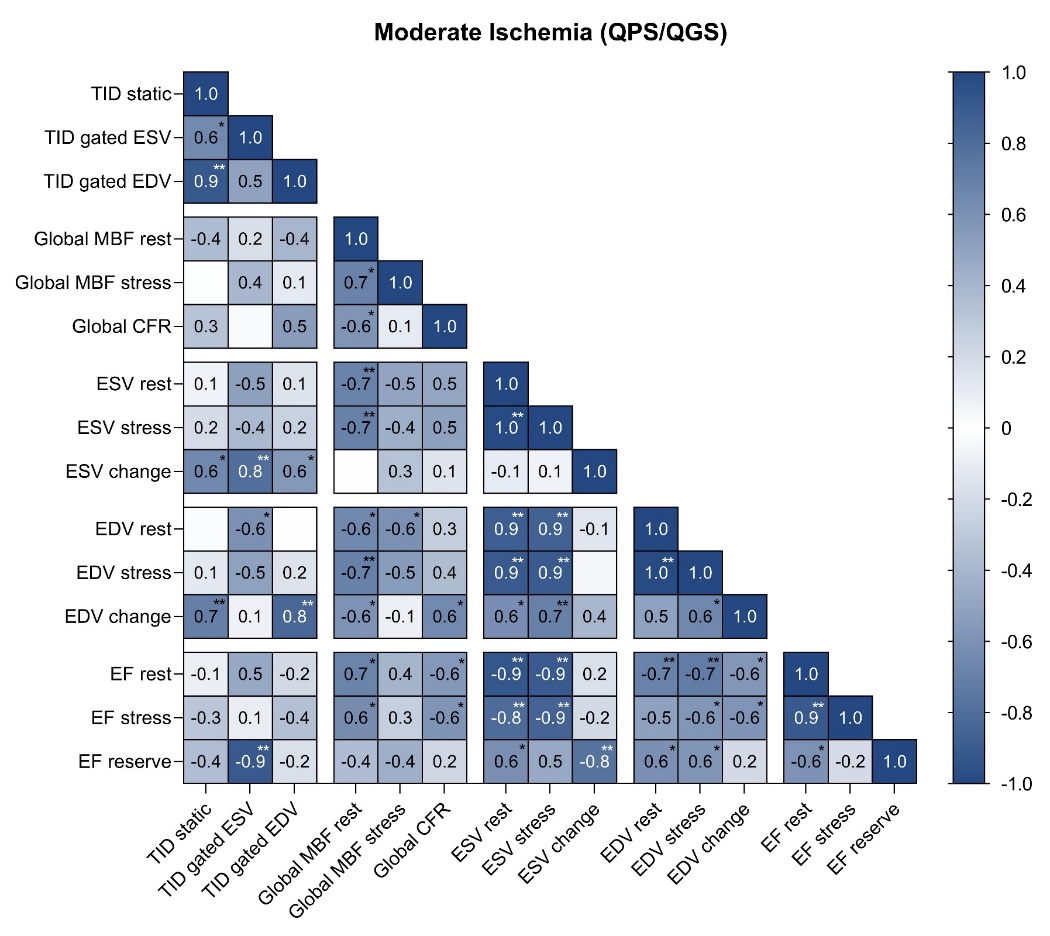

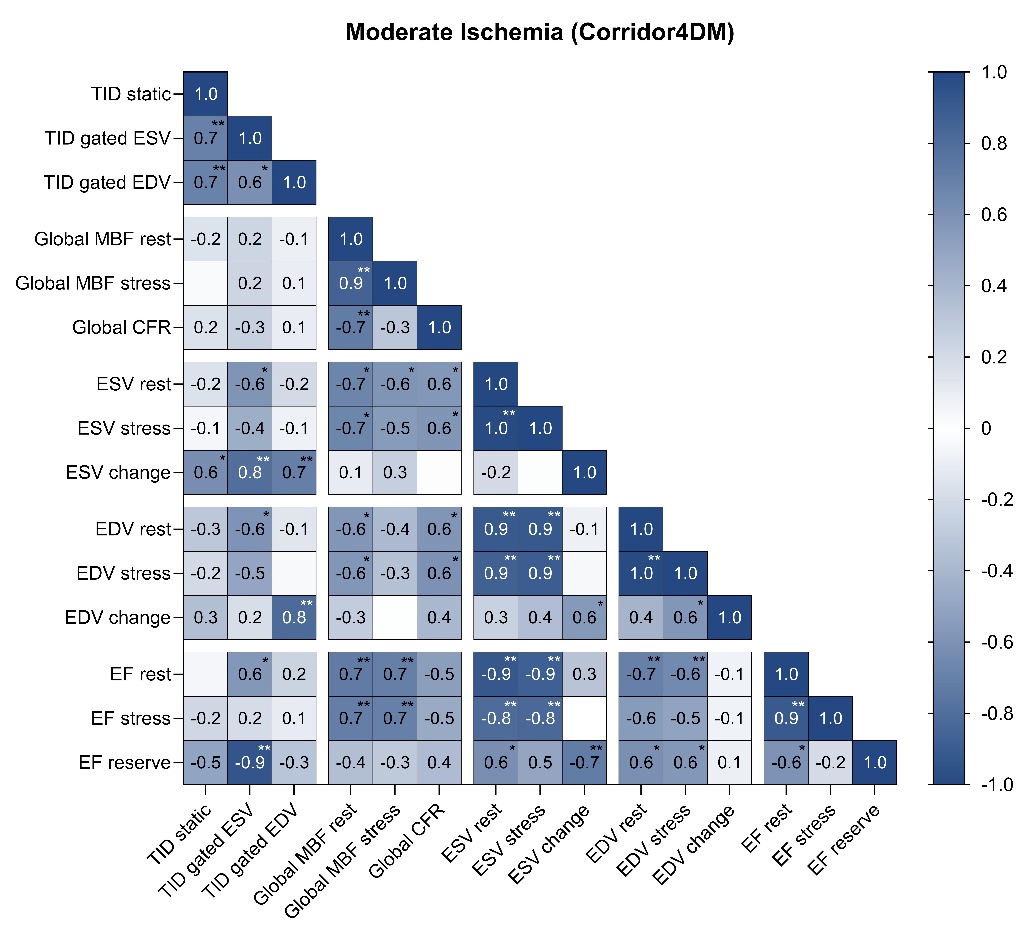


**a.**

**b.**

**Fig. 3** Correlation matrix depicting the relationships between TID ratios and parameters of LV function and perfusion from the moderate ischemic group. A. Analysis of values acquired with QPS/QGS. B. Analysis of values acquired with Corridor4DM. MBF, myocardial blood flow; CFR, coronary flow reserve; ESV, end-systolic volume; EDV, end-diastolic volume; EF, ejection fraction. **p*<0.5, ***p*<0.01.


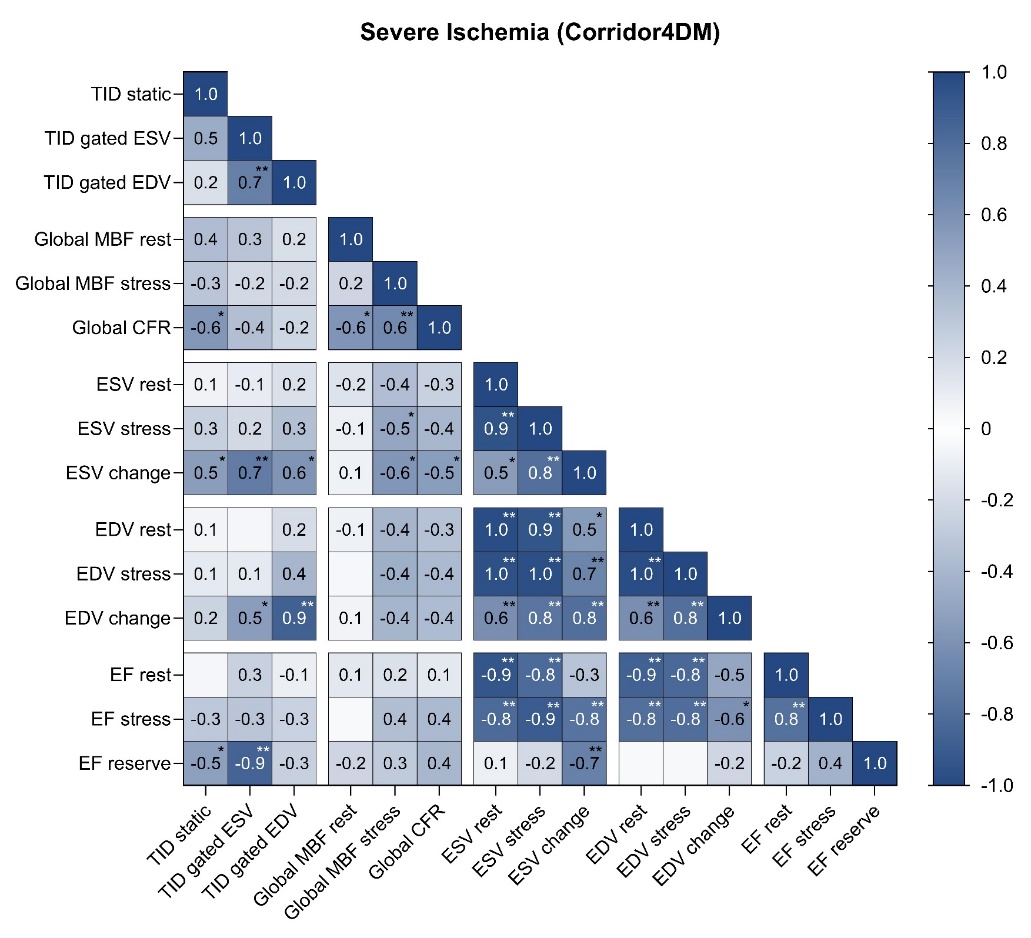

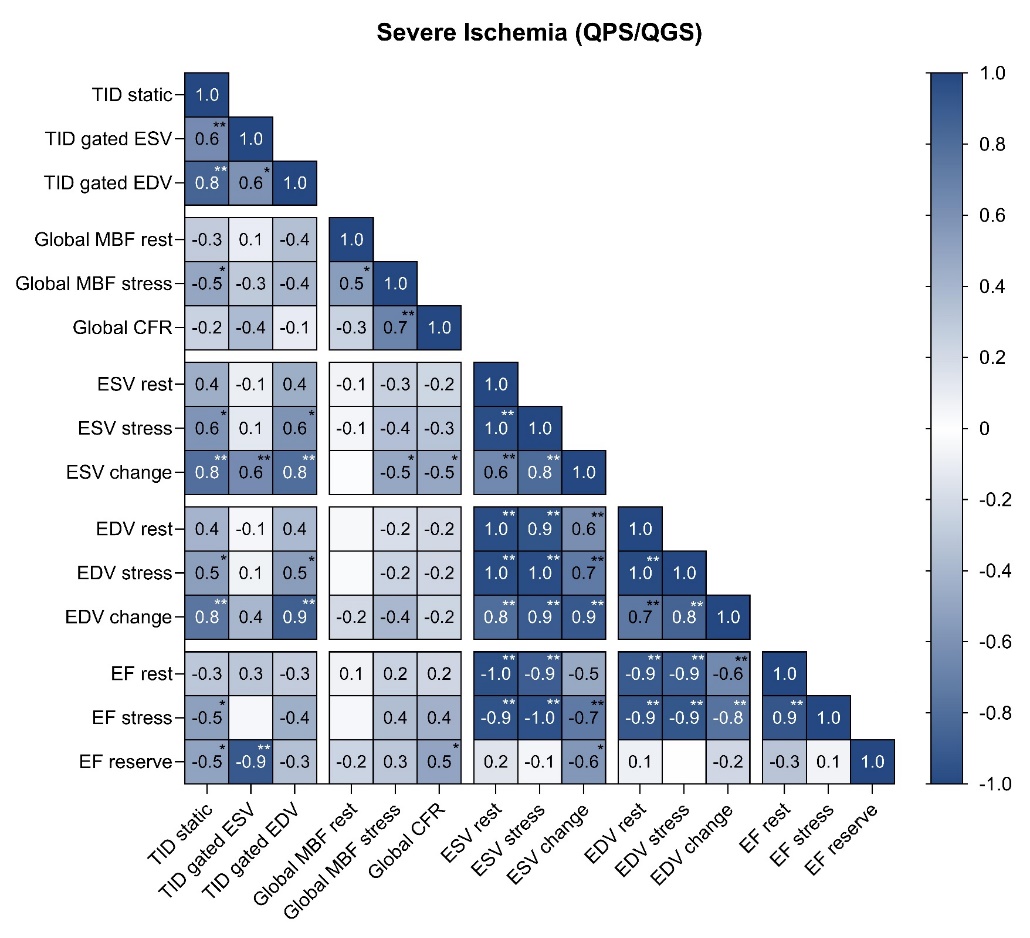


**a.**

**b.**

**Fig. 4** Correlation matrix depicting the relationships between TID ratios and parameters of LV function and perfusion from the severe ischemic group. A. Analysis of values acquired with QPS/QGS. B. Analysis of values acquired with Corridor4DM. MBF, myocardial blood flow; CFR, coronary flow reserve; ESV, end-systolic volume; EDV, end-diastolic volume; EF, ejection fraction. **p*<0.5, ***p*<0.01.


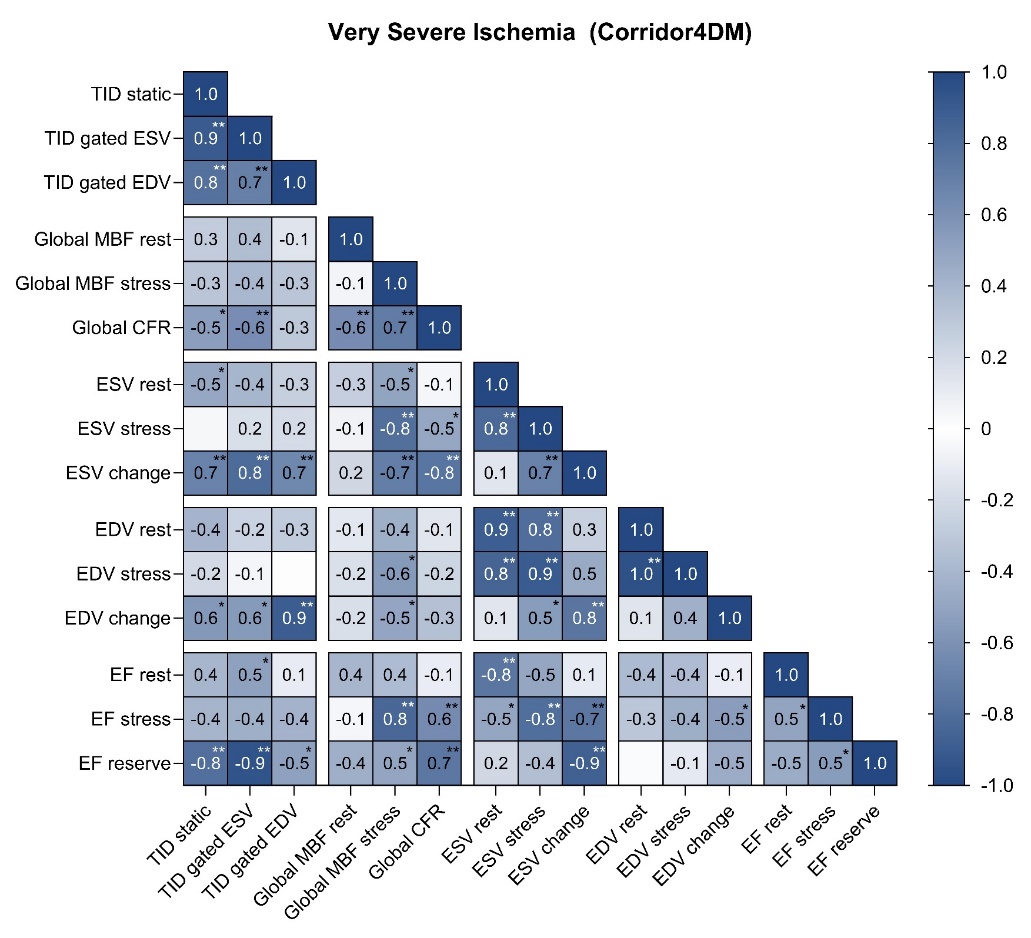

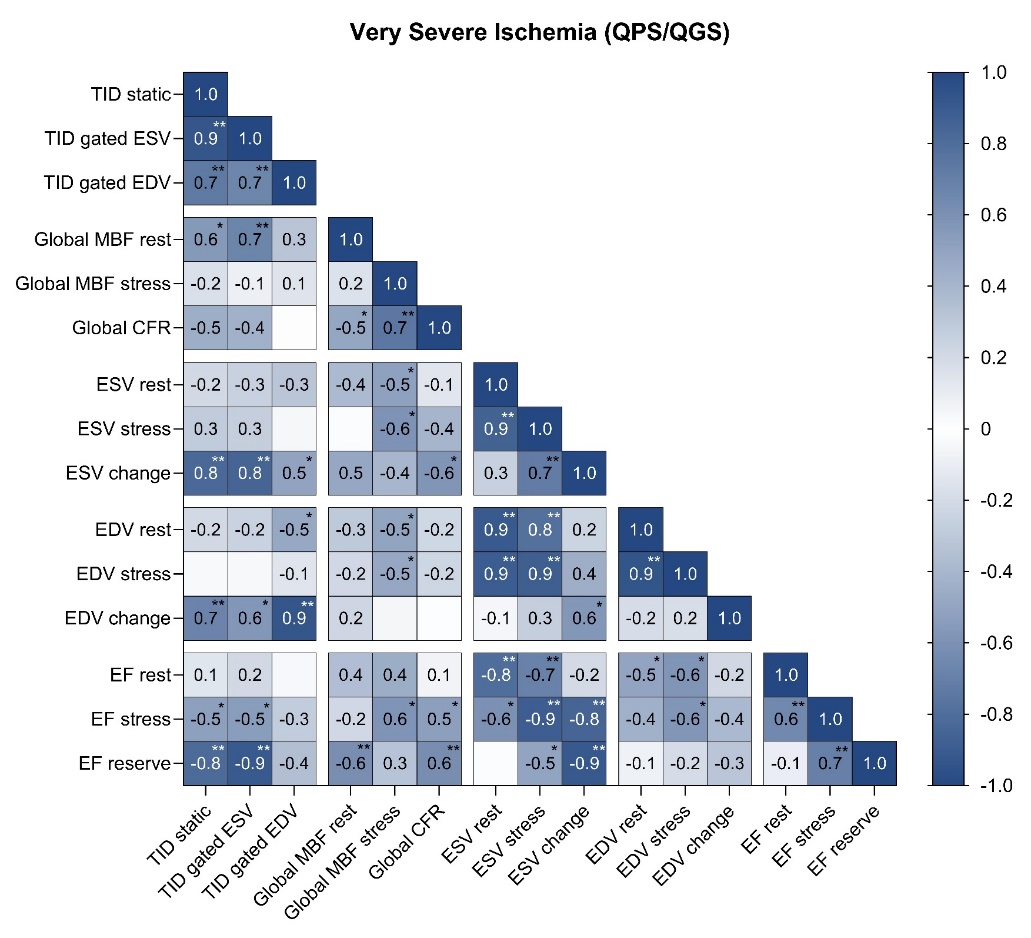


**a.**

**b.**

**B.**

**Fig. 5** Correlation matrix depicting the relationships between TID ratios and parameters of LV function and perfusion from the very severe ischemic group. A. Analysis of values acquired with QPS/QGS. B. Analysis of values acquired with Corridor4DM. MBF, myocardial blood flow; CFR, coronary flow reserve; ESV, end-systolic volume; EDV, end-diastolic volume; EF, ejection fraction. **p*<0.5, ***p*<0.01.
